# The Time-Varying Impact of COVID-19 on the Acute Kidney Disorders: A Historical Matched Cohort Study and Mendelian Randomization Analysis

**DOI:** 10.34133/hds.0159

**Published:** 2024-07-15

**Authors:** Chunyang Li, Chao Zhang, Jie Chen, Yilong Chen, Zhiye Ying, Yao Hu, Huan Song, Ping Fu, Xiaoxi Zeng

**Affiliations:** ^1^ Division of Nephrology, West China Biomedical Big Data Center, West China Hospital, Sichuan University, Chengdu 610041, China.; ^2^ Med-X Center for Informatics, Sichuan University, Chengdu 610065, China.; ^3^ Department of Core Laboratory, Sichuan Provincial Peopleʼs Hospital, University of Electronic Science and Technology of China, Chengdu 610054, China.; ^4^ Centre of Public Health Sciences, Faculty of Medicine, University of Iceland, Reykjavík, Iceland.

## Abstract

**Background:** This study aimed to explore the time-varying impact of COVID-19 on acute kidney disorders, including acute kidney injury and other acute kidney diseases. **Methods:** From the UK Biobank, 10,121 participants with COVID-19 were matched with up to 3 historically unexposed controls by age, sex, Townsend deprivation index, and the status of hospitalization or receiving critical care. We investigated the association between COVID-19 and incidence of acute kidney disorders, within the first 4 weeks after infection, using conditional and time-varying Cox proportional hazard regression. In addition, one-sample Mendelian randomization, utilizing the polygenic risk score for COVID-19 as an instrumental variable, was conducted to explore the potential causality of the association. **Results:** In the matched cohort study, we observed a significant association between COVID-19 and acute kidney disorders predominantly within the first 3 weeks. The impact of COVID-19 was time dependent, peaking in the second week (hazard ratio, 12.77; 95% confidence interval, 5.93 to 27.70) and decreasing by the fourth week (hazard ratio, 2.28; 95% confidence interval, 0.75 to 6.93). In subgroup analyses, only moderate to severe COVID-19 cases were associated with acute worsening of renal function in a time-dependent pattern. One-sample Mendelian randomization analyses further showed that COVID-19 might exert a “short-term” causal effect on the risk of acute kidney disorders, primarily confined to the first week after infection. **Conclusions:** The risk of acute kidney disorders following COVID-19 demonstrates a time-varying pattern. Hazard effects were observed only in patients with moderate or severe but not mild COVID-19.

## Introduction

The severe acute respiratory syndrome coronavirus 2 (SARS-CoV-2), which triggered COVID-19 pandemic, has significantly affected global health, resulting in approximately 7.0 million deaths worldwide by December 2023 [1]. COVID-19 affects both the respiratory system and extrapulmonary organs, such as the kidney [2], and exerts not only transient but also persistent influences on kidney function after infection [3]. Currently, the long-term consequences of COVID-19, an urgent public health priority [4,5], have attracted increasing attention.

In patients with COVID-19, kidney involvement is common. The incidence rate of acute kidney injury (AKI) can reach 41% to 62% [6–9] in patients with COVID-19. Despite the high incidence of AKI, research has revealed that the majority of patients can recover within the first week (the median number of days to recover from AKI is 6) following infection [10]. Studies also suggest that patients with COVID-19 with longer AKI recovery times have a higher incidence of chronic kidney disease (CKD) [11]. In addition, the impact of COVID-19 on kidney function has been observed to vary over time. A cohort study, in which 336,473 patients with COVID-19 were followed up for 96 weeks, showed that the incidence of AKI was highest in the first 16-week period (47%), decreased in the following 16 weeks (37%), and was relatively stable thereafter [12]. These findings indicate that COVID-19 might exert time-varying effects on kidney disorders.

Regarding the underlying mechanisms of kidney injury caused by COVID-19, in the consensus report of the 25th Acute Disease Quality Initiative Workgroup on COVID-19-associated AKI, both direct and indirect mechanisms have been proposed. Su et al. [5] reported direct infection of the kidney in a postmortem study. However, in other reports, no evidence of direct viral kidney invasion was found, and indirect effects caused by systemic effects of COVID-19 and critical care interventions, as well as organ cross-talk, might be other important mechanisms for AKI. Regarding other nephrologic conditions, such as glomerular disorders, a recent review summarized 76 studies on kidney pathology related to COVID-19 and revealed that although various glomerular diseases have been documented in patients with COVID-19, these lesions were typically identified when patients sought medical attention for issues unrelated to their kidney health [13], similar to other reports of cases of “SARS-CoV-2 nephropathy” [14]. Thus, whether SARS-CoV-2 directly causes kidney injury remains to be determined.

The current study aims to evaluate the time-varying associations between COVID-19 and acute kidney outcomes and to further assess their causal relationships. Using data from the UK Biobank, a population-based prospective cohort, we conducted a historical matched cohort study to investigate the “short-term” influence of COVID-19 on subsequent acute kidney disorders, including AKI and acute kidney diseases. Moreover, we performed one-sample Mendelian randomization (MR) to provide further insights into the causal links between COVID-19 and kidney diseases.

## Methods

### Observational, matched cohort study

#### Study population

From 2006 to 2010, the UK Biobank enrolled 500,000 participants, collecting extensive data on their genetic backgrounds, health statuses, and long-term follow-up results (see the Supplementary Materials for more details).

**Fig. 1.**
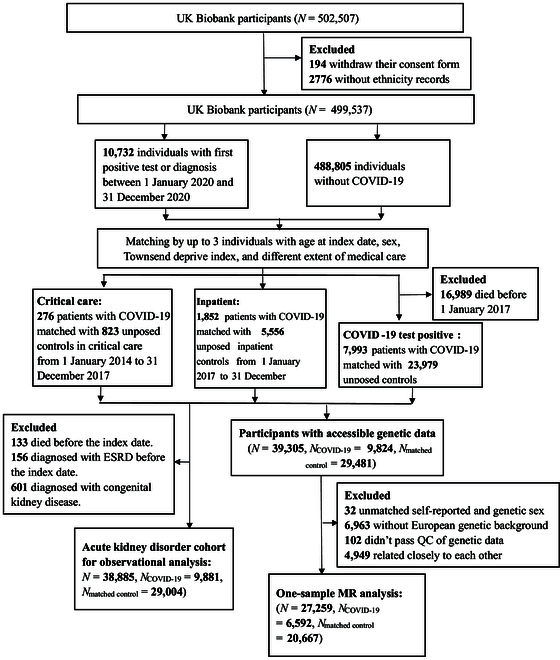
Flow chart of the study population selection of the acute kidney disorder cohort and CKD cohort. Up to 3 individuals without SARS-CoV-2 infection were randomly selected and individually matched to each infected individual by age at index date, sex, Townsend deprivation index, and severity of COVID-19. Finally, 38,885 and 27,259 participants were selected for observational and one-sample MR analyses, respectively. QC, quality control.

The exposed cohort included patients with positive SARS-CoV-2 tests or diagnostic codes for COVID-19 between 1 January 2020 and 31 December 2020 to eliminate the influence of the COVID-19 vaccine. Given the considerable proportion of undiagnosed individuals with COVID-19 in the whole population [15,16], we constructed a historically matched cohort using data collected prior to the pandemic. We firstly excluded individuals who withdrew their consent form (*n* = 194) or who did not report their ethnicities (*n* = 2,776). After matching exposed patients with COVID-19 with up to 3 unexposed historical controls, 40,479 participants were included in this matched cohort. Then, we further excluded participants if they (a) died before the index date (*n* = 133), (b) were diagnosed with end-stage renal disease before the index date (*n* = 156), and (c) were diagnosed with congenital kidney disease (*n* = 601). If the exposed COVID-19 cases were excluded, their corresponding unexposed controls were also excluded. Thus, 38,885 participants were ultimately included in the observational study. The flow chart of the study is shown in Fig. 1. This study followed the Strengthening the Reporting of Observational Studies in Epidemiology (STROBE) and Mendelian Randomization (STROBE-MR) reporting guidelines.

#### Identification of the COVID-19-exposed cohort and the matched unexposed cohort

The COVID-19-exposed cohort included the following: (a) mild cases with a positive SARS-CoV-2 test result ranging between 1 January 2020 and 31 December 2020, without hospitalization; (b) moderate cases, defined as hospitalized individuals diagnosed with COVID-19 (International Classification of Diseases 10th edition codes: U07.1 or U07.2) between 1 January 2020 and 31 December 2020 who did not fulfill the criteria for severe cases; and (c) severe COVID-19 cases, defined as individuals who received critical care within 7 d of diagnosis and/or undergoing invasive or noninvasive mechanical ventilation or other forms of respiratory support [Office of Population, Censuses and Surveys Classification of Surgical Operations and Procedures (OPCS) Classification of Interventions and Procedures version 4 codes listed in Table S1] [17].

The historically matched cohort was constructed by randomly selecting up to 3 unexposed individuals per patient with COVID-19 without replacement. We chose nonhospitalized individuals unexposed to COVID-19 to match with patients with mild COVID-19 and hospitalized patients admitted in 2017 to match patients with moderate COVID-19. For severe COVID-19, we matched unexposed inpatients who received invasive or noninvasive mechanical ventilation or other respiratory support treatments (codes listed in Table S1). Because of the relatively small sample size of patients with critical care records, we relaxed the admission date between 2014 and 2017 for the inclusion of critically ill controls. All pairs were matched by age (±3 years at index date), sex, and Townsend deprivation index (classified into low, medium, and high tertiles).

For the COVID-19-exposed cohort, the index date was designated as the date for COVID-19 diagnosis. The index date of the unexposed control for patients with mild COVID-19 was 3 years prior to that of his or her exposed counterpart (for example, if the index date of the positive SARS-CoV-2 infection was 2020 May 15, the index date of the matched historical control would be 2017 May 15) [17,18]. The index date of unexposed controls who were matched for moderate or severe COVID-19 was the admission date.

#### Ascertainment of outcomes

Diagnoses of acute kidney disorder were ascertained through linkage to hospital inpatient records (diagnoses and operations or procedures performed during inpatient admissions), national death registries, and the primary care system. In addition, we also linked EMIS codes used in the primary care system to the abovementioned International Statistical Classification of Diseases (ICD) codes using the Systematized Nomenclature of Medicine-Clinical Terms (SNOWMED CT) global standards for health terms [19]. EMIS codes with multiple matched ICD codes were further manually checked. The algorithmic end-stage renal disease (ESRD) definition was obtained from hospital admission records, self-reports verified by nurse interviews, or death certificate records [20].

In the present study, acute kidney disorder was defined as follows: (a) acute nephritic syndrome, rapidly progressive nephritic syndrome, acute tubulointerstitial nephritis, or acute renal failure (codes listed in Table S2) and (b) patients who underwent surgeries or procedures indicating receiving renal replacement therapy (Table S2). We excluded patients with ICD-9 or ICD-10 codes of congenital kidney disease for outcome identification (listed in Table S2). For the analysis of acute kidney disorders, the follow-up period of each participant was 28 d after the index date, the date of death, or the date of diagnosis, whichever occurred first.

#### Covariates

For covariates, we collected data on sex, body mass index, Townsend deprivation index, education level, income level, and smoking status at baseline, as well as age, comorbidities of hypertension, diabetes or CKD, and Charlson comorbidity index at the index date. More information about these covariates is described in the Supplementary Materials.

#### Statistical analyses

Continuous variables were expressed as medians with interquartile ranges (IQRs) and were compared by analysis of variance (ANOVA). Categorical variables were expressed as percentages and were compared by chi-square tests. The crude cumulative incidence was estimated from Kaplan–Meier survival curves [21]. In addition, to illustrate the time-varying effects of COVID-19, we assessed the instantaneous risk of acute kidney disorder in both the exposed and unexposed groups at any specific time point following infection. This was accomplished using the “bshazard” package, which offers a nonparametric, smoothed estimate of the hazard function via B-splines within the framework of generalized linear-mixed models [22–24]. We further analyzed the relative risk of acute kidney disease in the matched cohort using a conditional Cox proportional hazard regression model, allowing us to estimate hazard ratios (HRs) and their corresponding 95% confidence intervals (CIs). We adjusted the following predefined covariates in the multivariate model: age at index date (continuous), Townsend deprivation index (3 categories by quantile), sex (female or male), smoking status (never, previous, current, or unknown), body mass index (BMI) (thin, <18.5 kg/m^2^; normal, 18.5 ≤ BMI < 25 kg/m^2^; overweight, 25 ≤ BMI < 30 kg/m^2^; obese, ≥30 kg/m^2^; or unknown), ethnicity (white, Asian, black, mixed, or other or unknown), education level (college or university degree, A levels/AS levels/O levels/The General Certificate of Secondary Education (GCSEs)/GSEs or equivalent, other degree, or unknown), income level (ultrahigh, high, medium, low, or unknown), diabetes (presence or not), hypertension (presence or not), CKD (presence or not), and Charlson comorbidity index (0, 1, 2, or ≥3) (a detailed definition is listed in the Supplementary Materials). To evaluate the temporal patterns of the association between COVID-19 and the risk of incident acute kidney disorder, we divided 28-d follow-up time into 4 weeks. The relative risks at each time interval were reported as the HR and 95% CI using conditional Cox proportional hazard regression model.

#### Subgroup and sensitivity analyses

Subgroup analyses were performed according to sex and the severity of COVID- 19 (mild, moderate, or severe). To further test the robustness of our studies, sensitivity analyses of the observational studies were subsequently conducted. (a) For acute kidney disorder, the Fine and Gray [25] competing risk model in the “cmprsk” package was used to estimate subdistribution HRs, with death as the competing risk. (b) To further balance the characteristics between the exposed and unexposed groups, inverse probability weights based on age at index date, sex, ethnicity, Townsend deprivation index, BMI, smoking status, education level, income level, diabetes, hypertension, CKD, and Charlson comorbidity index were firstly measured [26]. Then, univariate Cox proportional hazard regression was performed after weighting. The standard mean difference (SMD) between the exposed and unexposed groups is described in Table S3, and an SMD of <0.2 indicated a sufficient balance between the 2 groups [27]. (c) We further restricted the outcome of AKI (defined as the ICD-10 code N17 from hospital inpatient records and the death registry) and evaluated the time-varying effects of COVID-19 on the subsequent risk of incident AKI using conditional (for the matched cohort) and univariate (for postweighting) Cox proportional hazard regression models.

### MR analyses

#### Study population

We conducted one-sample MR analyses to further evaluate the short-term causal impact of COVID-19 on the risk of subsequent kidney disorders. On the basis of the matched cohort (*N* = 40,479), we further selected participants with accessible genetic data (*n* = 39,305) and excluded participants with unmatched self-reported and genetic sex (*n* = 32), without a European genetic background (*n* = 6,963), who did not pass quality control of genetic data (*n* = 102), and who were closely related to each other (with a kinship coefficient over 0.0884, *n* = 4,949) [28], which totally leveraging 27,259 participants in one-sample MR study (Fig. 1).

#### One-sample MR for causal association between COVID-19 and kidney disease

Polygenic risk scores of mild to severe COVID-19 in the abovementioned 27,259 participants who served as exposures were firstly calculated, with genome-wide association studies summary statistics of COVID-19 susceptibility (defined as mild COVID-19), hospitalization (defined as moderate COVID-19), and severity (defined as severe COVID-19) derived from the COVID-19 Host Genetics Initiative (r7) database as targeted data [29] (listed in Table S4).

One-sample MR analyses were executed through a 2-step sequential regression method. Initially, a logistic regression model was used to analyze the effect of exposure variables—scaled polygenic risk scores for mild, moderate, and severe COVID-19—against the outcome of COVID-19. This model was adjusted for continuous age, sex (defined as male or female), and the first 10 genetic principal components (continuous variables) [30]. In the subsequent stage, the scaled predicted exposure values from the first step, which indicate COVID-19 phenotypes, were analyzed for their association with the presence of acute kidney disorder using a Cox proportional hazards regression model. Additional adjustments were made for BMI, smoking status, hypertension, diabetes, and the Charlson comorbidity index. The initial MR estimate (β) was calculated as the change in the log odds for acute kidney disorder linked with a one-unit increase in the log odds of COVID-19. To facilitate interpretation, this estimate was then scaled to represent the effect per doubling in the odds of COVID-19 by multiplying by 0.693 [31].

Data were analyzed using R software (version 4.1.2) and PLINK (version 1.9). A *P* < 0.05 was considered as a possible association between COVID-19 and acute kidney disorder.

## Results

### Characteristics of the matched cohort

The observational cohort for evaluating the association between COVID-19 and acute kidney disorders included a total of 9,881 infected individuals, matched with 29,004 uninfected participants (Table 1 and Fig. 1). The mean ages were 64.68 and 65.13 years for exposed and unexposed individuals, and 47.72% and 47.43% were male in these 2 groups, respectively. As shown in Table 1, a lower proportion of participants with COVID-19 infections had college education, and a higher proportion had obesity, hypertension, diabetes, or CKD at baseline.

**Table 1.** Characteristics of patients with COVID-19 and matched controls

	Acute kidney disorders (*N* = 38,885)		
Baseline characteristics	COVID-19 (*N* = 9,881)	Unexposed controls^a^ (*N* = 29,004)	*P* value
Follow-up time, d	28 (28–28)	28 (28–28)	<0.001
Age at index, years	64.68 (58.35–72.90)	65.13 (58.62–72.77)	0.4
Townsend deprivation index^b^			0.9
High	4,033 (40.82%)	11,817 (40.74%)	
Medium	3,131 (31.69%)	9,202 (31.73%)	
Low	2,717 (27.50%)	7,985 (27.53%)	
Sex			0.6
Female	5,166 (52.28%)	15,246 (52.57%)	
Male	4,715 (47.72%)	13,758 (47.43%)	
Smoking status			0.1
Current	1,173 (11.87%)	3,326 (11.47%)	
Previous	3,508 (35.50%)	10,015 (34.53%)	
Never	5,165 (52.27%)	15,541 (53.59%)	
Unknown	35 (0.35%)	120 (0.41%)	
Education level			<0.001
College or university degree	2,489 (25.19%)	9,100 (31.37%)	
A levels /AS levels /O levels / GCSEs / GSEs or equivalent	4,073 (41.22%)	10,841 (37.38%)	
Other qualifications	1,256 (12.71%)	3,350 (11.55%)	
Unknown	2,063 (20.88%)	5,713 (19.70%)	
Income level			<0.001
Ultra high	447 (4.52%)	1,308 (4.51%)	
High	1,741 (17.62%)	4,992 (17.21%)	
Medium	4,384 (44.37%)	12,287 (42.36%)	
Low	2,929 (19.52%)	5,996 (20.67%)	
Unknown	1,380 (13.97%)	4,421 (15.24%)	
BMI			<0.001
<18.5	37 (0.37%)	150 (0.52%)	
18.5 ≤ BMI < 25	2,621 (26.53%)	8,953 (30.87%)	
25 ≤ BMI < 30	4,076 (41.25%)	12,419 (42.82%)	
≥30	3,081 (32.18%)	7,318 (25.23%)	
Unknown	66 (0.67%)	164 (0.57%)	
Hypertension at index date			0.02
Yes	3,744 (37.89%)	10,618 (36.61%)	
No	6,137 (62.11%)	18,386 (63.39%)	
Diabetes at index date			<0.001
Yes	1,175 (11.89%)	2,604 (8.98%)	
No	8,706 (88.11%)	26,400 (91.02%)	
CKD at index date			<0.001
Yes	625 (6.33%)	1,323 (4.56%)	
No	9,256 (93.67%)	27,681 (95.44%)	
Charlson comorbidity index^c^			<0.001
0	6,120 (61.94%)	20,080 (69.23%)	
1	1,552 (15.71%)	3,910 (13.48%)	
2	979 (9.91%)	2,520 (8.69%)	
≥3	1,230 (12.45%)	2,494 (8.60%)	
Ethnicity			<0.001
White	9,007 (91.15%)	27,199 (93.78%)	
Asian or Asian British	411 (4.16%)	777 (2.68%)	
Black or Black British	252 (2.55%)	569 (1.96%)	
Mixed	77 (0.78%)	158 (0.54%)	
Other	134 (1.36%)	301 (1.04%)	
Acute kidney disorders			<0.001
Yes	465 (4.71%)	288 (0.99%)	
No	9,416 (95.29%)	28,716 (99.01%)	
COVID-19 diagnosis status			0.7
Severe COVID-19	263 (2.66%)	728 (2.51%)	
Moderate COVID-19	1,770 (17.91%)	5,160 (17.79%)	
Mild COVID-19	7,848 (79.43%)	23,116 (79.70%)	

The values were reported as median (IQR) for continuous variables or number (%) for categorical variables.

^a^ Up to 3 uninfected individuals were randomly selected and matched with per patient with COVID-19 individually by age at index date, sex, Townsend deprivation index, and severity of COVID-19.

^b^ Townsend deprivation index was assigned to each individual and categorized into tertiles. A greater index score implies a greater degree of deprivation.

^c^ Charlson comorbidity index at the index date, calculated on the basis of UK Biobank hospital episode data (see the Supplementary Materials).

### Associations between COVID-19 and subsequent kidney traits in observational study

**Fig. 2.**
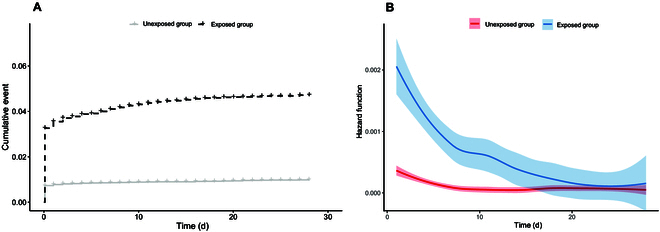
Effect of COVID-19 on the cumulative incidence and smoothed hazard function of acute kidney disorder. (A) The cumulative incidence of acute kidney disorder in exposed (black) and unexposed (gray) participants during follow-up. (B) Hazard function smoothed with B-spline depicting the instantaneous risk of COVID-19 on acute kidney disorder in exposed (blue) and unexposed (red) participants during follow-up. The dark blue/red lines represent the estimated hazard functions with 95% CIs in the light blue/red regions.

**Fig. 3.**
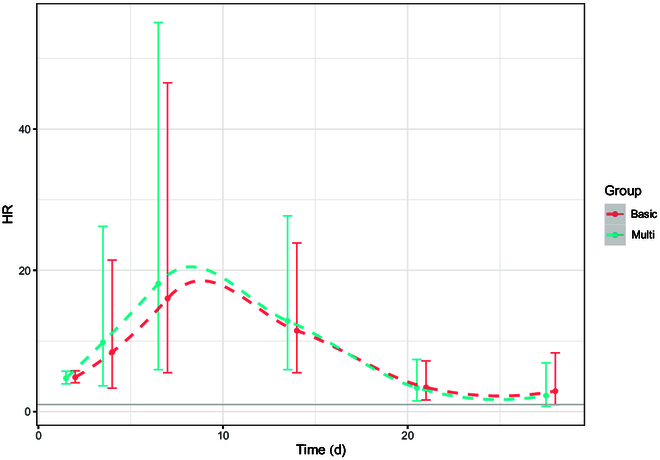
Temporal changes in the association between COVID-19 and subsequent acute kidney disorder. The simulated curves were smoothed using one-dimensional smoothing spline. Basic model adjusted for age. The multivariable model was additionally adjusted for BMI, hypertension, diabetes, CKD, smoking status, education level, income level, and Charlson comorbidity index.

For the evaluation of the short-term impact of COVID-19 on the kidney, we observed 465 and 288 acute kidney disorder cases in the exposed and matched control groups, corresponding to a crude incidence rate of 1.8 and 0.36 per 1,000 person-days, respectively (Table 2). As shown in Fig. 2A, the cumulative incidence was significantly different between the exposed and matched control groups. The absolute risk for incident acute kidney disorder in the exposed group changed over time during the 28-d follow-up; specifically, it seemed much higher in approximately the first 20 d after infection (Fig. 2B). The average HR for acute kidney disorder was 5.16 (95% CI, 4.35 to 6.11) in the whole surveillance period, while the *P* value for the PH test was less than 0.05, indicating that the proportional hazard assumption was violated (Table 2). Therefore, we performed a time-varying analysis. As shown in Fig. 3 and Table 3, the magnitude of the association between COVID-19 and acute kidney disorder increased at first, reaching a peak at the second week, and then gradually decreased with no significance in the fourth week after infection. The corresponding HRs for the first to the fourth week were 5.08 (95% CI, 4.24 to 6.08), 12.77 (95% CI, 5.93 to 27.70), 3.38 (95% CI, 1.54 to 7.41), and 2.28 (95% CI, 0.75 to 6.93), respectively. When stratified by sex, COVID-19 had similar temporal effects on acute kidney disorder in both males and females, but a nonsignificant association was observed in the third week for males and in the fourth week for females (Table 3). Interestingly, severe COVID-19 only had hazard effects on the risk of acute kidney disorder incidence in the first week, while mild COVID-19 infection had no significant influence on the subsequent risk of incident acute kidney disorder over the 4 weeks (Table 3).

**Table 2.** Association of COVID-19 with the risk of acute kidney disorder incidence using observational analysis

No. of cases per 1,000 person-days		Basic model^a^			Multi-variable model^b^		
Exposed	Matched controls	HR (95% CI)	*P* value	pH assumption test for COVID-19	HR (95% CI)	*P* value	pH assumption test for COVID-19
465/1.8	288/0.36	5.23 (4.47–6.12)	<0.001	0.01	5.16 (4.35–6.11)	<0.001	0.004

^a^ Basic model adjusted for age at index date.

^b^ Multivariable model additionally adjusted for BMI, hypertension, diabetes, CKD, smoking status, education level, income level, and Charlson comorbidity index.

**Table 3.** Time-varying effects of COVID-19 on acute kidney disorder and subgroup analysis

	No. of cases per 1,000 person-day		The first week (days 0 to 7)		The second week (days 8 to 14)		The third week (days 15 to 21)		The fourth week (days 22 to 28)	
	Exposed	Matched controls	HR (95% CI)	*P* value	HR (95% CI)	*P* value	HR (95% CI)	*P* value	HR (95% CI)	*P* value
**Primary analysis** ^a^	465/1.8	288/0.36	5.08 (4.24– 6.08)	<0.001	12.77 (5.93–27.70)	<0.001	3.38 (1.54–7.41)	0.002	2.28 (0.75– 6.93)	0.2
**Subgroup analysis by stratified of sex** ^b^
Male	301/2.5	203/0.54	4.93 (3.95– 6.15)	<0.001	9.89 (4.23– 23.13)	<0.001	2.24 (0.82– 6.14)	0.1	3.65 (0.80– 16.77)	0.1
Female	164/1.19	85/0.20	5.91 (4.26– 8.19)	<0.001	46.06 (5.48– 387.25)	<0.001	5.80 (1.44–23.29)	0.01	1.07 (0.18– 6.55)	0.9
**Subgroup analysis by stratified of Severity of the disease** ^c^
Severe COVID-19	110/32.2	154/12.3	2.47 (1.83– 3.33)	<0.001	1.48 (0.12– 17.23)	0.8	2.25 (0.34– 15.11)	0.4	NA	NA
Moderate COVID-19	346/9.18	124/0.88	7.34 (5.71– 9.44)	<0.001	22.21 (8.18– 60.27)	<0.001	6.13 (1.77– 21.26)	0.004	2.26 (0.47–7.66)	0.2
Mild COVID-19	9/0.04	10/0.02	NA	NA	1.05 (0.01– 84.88)	0.9	3.59 (0.48– 26.76)	0.2	4.86 (0.05– 505.37)	0.5

Covariates adjusted in the model including age, BMI, hypertension, diabetes, CKD, smoking status, education level, income level, and Charlson comorbidity index.

^a^ The reference here is unexposed individuals in the cohort.

^b^ The reference here is unexposed male or female individuals in the cohort, respectively.

^c^ The reference here is unexposed severe, moderate, or mild individuals in the cohort, respectively.

In sensitivity analysis of the observational study, the results did not change. Briefly, when death was considered a competing risk, the highest HR for acute kidney disorder was still present in the second week and disappeared in the fourth week after infection (Table S5). The time-varying effect of COVID-19 on subsequent incident acute kidney disorder did not change after weighting, peaking at the second week and remaining nonsignificant at the fourth week (Table S5). When we further restricted the outcome of AKI using the diagnostic code of N17 (458 and 240 patients in the exposed and their matched control groups, respectively), the time-varying effects of COVID-19 on the subsequent risk of incident AKI remained both in the pre- and postweighting analyses (Table S6).

### Causal effects of COVID-19 on kidney traits in one-sample MR analysis

Genetically predicted mild to severe COVID-19 might be associated with incident acute kidney disorder risk only in the first week after infection. For example, per doubling the odds of severe COVID-19 might be associated with a 79% increased risk of acute kidney disorder incidence (odds ratio, 1.79; 95% CI, 1.36 to 2.35; *P* < 0.001) (as shown in Table 4).

**Table 4.** One-sample MR results between COVID-19 and the risk of incident acute kidney disorder

	The first week (days 0 to 7)		The second week (days 8 to 14)		The third week (days 15 to 21)		The fourth week (days 22 to 28)	
	HR (95% CI)	*P* value	HR (95% CI)	*P* value	HR (95% CI)	*P* value	HR (95% CI)	*P* value
**Severe COVID-19**	11.48 (4.12–31.98)	<0.001	470.42 (7.45–29716.98)	0.004	4.71 (0.01–1484.51)	0.60	0.04 (1.34 × 10^−5^–105.46)	0.42
**Moderate COVID-19**	3.68 (1.13–11.95)	0.03	46.41 (0.55–3922.54)	0.09	7.75 (0.01–4248.00)	0.52	20.67 (0.003–1.24×10^5^)	0.50
**Mild COVID-19**	2.71 (1.31–5.61)	0.007	12.02 (0.88–164.83)	0.06	0.02 (0.0003–0.95)	0.05	82.08 (1.18–5688.88)	0.05

The reference here is unexposed severe, moderate, or mild individuals in the cohort, respectively.

## Discussion

In this matched cohort study, we observed a temporal association between COVID-19 and subsequent acute kidney disorder, and the time-dependent hazard effects were detected exclusively in patients with moderate to severe COVID-19 but not in patients with mild COVID-19. Consistently, genetically predicted mild, moderate, and severe COVID-19 might also have a short-term influence on the subsequent risk of acute kidney disorder incidence. A series of sensitivity analyses supported the same conclusion from the primary analysis. Our study provides evidence for the time-varying influences of COVID-19 on subsequent acute kidney disorder.

First, our findings of the time-varying effects of COVID-19 on the risk of incident acute kidney disorder are novel. Previous studies reported that patients with COVID-19 had a greater risk of AKI incidence [32], higher overall mortality, and less AKI recovery than patients without COVID-19 [32,33]. SARS-CoV-2 infection impairs kidney function through acute tubular injury, viral cell invasion, and vascular damage [34], leading to increased serum creatinine, hematuria, and/or proteinuria levels [35]. However, few studies have focused on the temporal influence of COVID-19 on acute kidney disorder. We observed that the risk of acute kidney disorder reached its highest level in the second week after the initiation of COVID-19 and then gradually decreased with no significance in the fourth week. Consistent with a previous study reporting that organ dysfunction tends to occur in the second week after SARS-CoV-2 infection [36], the median time to AKI occurrence was approximately 8 to 9 d after infection, corresponding to the second week [37,38]. Although comparable results of the time-varying hazard effects of COVID-19 on the risk of subsequently kidney outcomes are lacking, one previous study reported similar temporal trends of COVID-19 on subsequent life-threatening secondary infections [18]. To our knowledge, this is the first study to describe the association between COVID-19 and acute kidney disorder over time.

Second, a major strength of our study is that the time-varying risks of developing acute kidney disorder were significant only among patients with severe or moderate COVID-19, but not among patients with mild COVID-19. Considering that the severity of COVID-19 might influence kidney function differentially, we matched uninfected controls with patients with COVID-19 according to different extents of medical care: nonhospitalization, hospitalization without critical care, and critical care with respiratory support treatments, to balance clinical or demographic characteristics for each matched group. An observational analysis showed similar results that severe COVID-19 only significantly affected the risk of incident acute kidney disorder in the first week, but not in the next 3 weeks. Consistent with previous studies, patients with no AKI diagnosis had the highest transition rates to stage 1 AKI within the first week during the whole intensive care unit stay [39]. The incidence rate of AKI in patients with severe COVID-19 ranges from 41% to 62% [6–8], and most cases are classified as transient AKI that rapidly develops within the first week after SARS-CoV-2 infection [40,41]. Furthermore, we observed that mild COVID-19 infection had no significant influence on the risk of subsequent acute kidney disorder over the 4 weeks. One previous study observed that among 58 patients with mild to moderate COVID-19, no AKI was identified [42], with a low incidence of AKI in patients with mild to moderate COVID-19. This result is consistent with our observational findings that only 9 acute kidney disorder cases were observed among patients with mild COVID-19, corresponding to a crude incidence rate of 0.04 per 1,000 person-days. Another study reported a 4.5% incidence of AKI in 66 hospitalized patients with COVID-19, indicating a relatively lower incidence of AKI in patients with mild to moderate COVID-19 [42]. Although the underlying mechanisms of COVID-19 and subsequent acute kidney disorder remain unclear, it could be speculated that COVID-19 might be a direct (such as a change in renal blood flow, toxicity, ischemic injury, or cytokine storm) [43] or indirect (such as binding with angiotensin-converting enzyme-2 highly expressed in the proximal tubule of the kidney) cause of kidney function change, and some debates still exist about the its causal relationship [44]. Our study provided evidence that prevention of kidney function deterioration in patients with severe or moderate SARS-CoV-2 infection was crucial.

In conclusion, our study utilized both observational and one-sample MR approaches to systematically investigating the causal relationship between COVID-19 and various kidney diseases. While randomized controlled trials are the benchmark for causal inference, MR offers a valuable alternative method that is both time-efficient and cost-effective [30]. Furthermore, we applied extensive and complementary sensitivity analyses to address potential confounders due to pleiotropy. The findings from these sensitivity analyses were largely robust and reliable.

However, our study has several limitations. First, our study was limited to participants of European descent, meaning that the applicability of these findings to other ethnic groups needs further exploration. Second, the SARS-CoV-2 has evolved over time, from alpha, beta, delta to omicron. We could not exactly differentiate the types of infected viruses in the publicly available genome-wide association studies, and further studies should be performed. Third, although historical controls were matched to eliminate the impact of undiagnosed COVID-19 cases, historical controls might differ from the exposed group due to the change of disease spectrum, and deeper analysis should be performed when more data are available. Fourth, because of the limitation of the data, we recruited only patients with COVID-19 diagnosed in 2020, a small sample size of the study population, which might lead to variability bias in the results. One-sample MR analysis found the trend that mild to severe COVID-19 might be associated with an increased risk of acute kidney disorder incidence in the first week, but the HR in the one-sample MR analysis should be interpreted with caution due to the limited sample size. Last, the underlying molecular mechanisms linking COVID-19 to subsequent acute kidney disorder are still unclear, and further investigations may be needed to further explore the causality.

### Conclusion

The impact of COVID-19 on the subsequent short-term acute kidney disorder was time dependent; the magnitude increased at first reaching a peak at the second week and then gradually decreasing with no significance in the fourth week after infection. In subgroup analyses, time-dependent effects were detected exclusively in patients with moderate to severe COVID-19, but not in patients with mild COVID-19. One-sample MR analyses further showed that COVID-19 might exert a “short-term” causal effect on the risk of acute kidney disorders, primarily confined to the first week after infection.

## Ethical Approval

All the UK Biobank participants provided written informed consent before data collection. The UK Biobank has full ethical approval from the National Health Service National Research Ethics Service (16/NW/0274). This study was approved by the Biomedical Research Ethics Committee of West China Hospital (2019-1171).

## Data Availability

The data underlying this article can be applied from the UK Biobank (http://www.ukbiobank.ac.uk/register-apply). The publicly available summary statistics of COVID-19 can be downloaded from the Host Genetics Initiative database (https://www.covid19hg.org/results/r7/).
