# Supplementary material for: The Time-Varying Impact of COVID-19 on the Acute Kidney Disorders: A Historical Matched Cohort Study and Mendelian Randomization Analysis
Source: Health Data Sci. 2024 Jul 15;4:0159. doi: 10.34133/hds.0159 (PMC11246837; doi:10.34133/hds.0159)
Supplement: Supplementary 1 — Supplementary Text Tables S1 to S6 [file hds.0159.f1.zip › Supplementary Materials-total.docx]

**Supplementary Materials**

1. **UK Biobank**

UK Biobank recruited about 500,000 participants aged 40–69 years from the UK general population between 2006 and 2010, and provides information, including in depth genetic backgrounds, health status, and outcomes of long- term follow-ups (Supplementary Materials) 15. Health-related outcomes were obtained through regular linkage with death, hospital inpatient, cancer and primary care (GP) records. For example, data on hospital admissions were obtained from Hospital Episode Statistics for England, Scottish Morbidity Record for Scotland, and Patient Episode Database for Wales. Death data were obtained from linkage to National Health Service (NHS) England for England and Wales, NHS Central Register, National Records of Scotland for Scotland. Individuals from UK Biobank was further linked to the primary care (GP) data from The Phoenix Partnership (TPP) for England and Egton Medical Information Systems (EMIS) Health GP system for Scotland and Wales 16. The results of SARS-CoV-2 test for COVID-19 were obtained from Public Health England (PHE) (England), Public Health Scotland (PHS) (Scotland) and Secure Anonymized Information Linkage (SAIL) (Wales).

1. **Ascertainment of diabetes for covariates**

Diabetes were defined based on 1) self-reported diabetes (See below) 2) inpatient diagnosis of diabetes (ICD-9 code of 250 and ICD-10 codes of E10, E11, E12, E13, E14) ^1^.

| **Number** | **Descriptions** | **Questions** | **Positive answers in UK Biobank** |
| --- | --- | --- | --- |
| 1 | Diabetes diagnosed by doctor | Has a doctor ever told you that you have diabetes? | “Yes” |
| 2 | Non-cancer illness code, self-reported |  | Code of 1220, 1222 or 1223 |
| 3 | Medication for cholesterol, blood pressure, diabetes, or take exogenous hormones | “Do you regularly take any of the following medications? (You can select more than one answer)” | “Insulin” |
| 4 | Medication for cholesterol, blood pressure or diabetes | “Do you regularly take any of the following medications? (you can select more than one answer)” | “Insulin” |

1. **Ascertainment of hypertension for covariates**

Hypertension were defined based on 1) self-reported hypertension (See below) 2) inpatient diagnosis of hypertension (ICD-9 code of 401, 402, 403, 404 and 405, and ICD-10 codes of I10, I11, I12, I13 and I15) ^1^.

| **Number** | **Descriptions** | **Questions** | **Positive answers in UK Biobank** |
| --- | --- | --- | --- |
| 1 | Vascular/heart problems diagnosed by doctor | “Has a doctor ever told you that you have had any of the following conditions? (You can select more than one answer)” | “High blood pressure” |
| 2 | Non-cancer illness code, self-reported |  | Code of 1065, 1072 |
| 3 | Medication for cholesterol, blood pressure, diabetes, or take exogenous hormones | “Do you regularly take any of the following medications? (You can select more than one answer)” | “Blood pressure medication” |
| 4 | Medication for cholesterol, blood pressure or diabetes | “Do you regularly take any of the following medications? (you can select more than one answer)” | “Blood pressure medication” |
| 5 | Age high blood pressure diagnosed | “What was your age when the high blood pressure was first diagnosed?” | Had records at initial assessment visit (2006-2010) at which participants were recruited and consent given |

1. **Ascertainment of Charlson comorbidity index for covariates**

Charlson comorbidity index at the index date for each participant was calculated according to diagnoses of the UK Biobank inpatient hospital data, which designated the index of baseline comorbidity levels ^2, 3^.ICD-10 codes for Charlson comorbidity index were listed below:

| **Score** | **Comorbidity** | **ICD-10 codes** |
| --- | --- | --- |
| 1 | Myocardial infarction | I21, I22, I25.2 |
| 1 | Congestive heart failure | I11.0, I13.0, I13.2, I50 |
| 1 | Peripheral vascular disease | I70, I71, I73.1, I73.8, I73.9, I77.1, I79.0, I79.2, K55.1, K55.8, K55.9, R02, Z95.8, Z95.9 |
| 1 | Cerebrovascular disease | G45, G46, I60, I61, I62, I63, I64, I65, I66, I67, I68, I69 |
| 1 | Dementia | F00, F01, F02, F03, F051, G30, G31.1 |
| 1 | Chronic pulmonary disease | J60, J61, J62, J63, J64, J65, J66, J67, J68.4, J70, J84.1, J92.0, J96.1, J98.2, J40, J41, J42, J43, J44, J45, J46, J47 |
| 1 | Connective tissue disease | M05, M06, M30, M31.5, M32, M33, M34, M35.1, M35.3, M36.0 |
| 1 | Ulcer disease | K25, K26, K27, K28 |
| 1 | Mild liver disease | B18, K70.0, K70.1, K70.2, K70.3, K70.9, K71.3, K71.4, K71.5, K71.7, K73, K74, K76.0, K76.2, K76.3, K76.4, K76.8, K76.9 |
| 1 | Diabetes mellitus | E10.0, E10.1, E10.6, E10.8, E10.9, E11.0, E11.1, E11.6, E11.8, E11.9, E12.0, E12.1, E12.6, E12.8, E12.9, E13.0, E13.1, E13.6, E13.8, E13.9, E14.0, E14.1, E14.6, E14.8, E14.9 |
| 2 | Hemiplegia | G04.1, G11.4, G80.1, G80.2, G81, G82, G83.9, G83.0, G83.1, G83.2, G83.3, G83.4 |
| 2 | Moderate/severe renal disease | I12.0, I13.1, N03.2, N03.3, N03.4, N03.5, N03.6, N03.7, N05.2, N05.3, N05.4, N05.5, N05.6, N05.7, N18, N19, N25.0, Z94.0, Z99.2 |
| 2 | Diabetes mellitus with chronic complications | E10.2, E10.3, E10.4, E10.5, E10.7, E11.7, E12.7, E13.7, E14.7, E11.2, E11.3, E11.4, E11.5, E12.2, E12.3, E12.4, E12.5, E13.2, E13.3, E13.4, E13.5, E14.2, E14.3, E14.4, E14.5 |
| 2 | Any tumor | C97, C43, C00, C01, C02, C03, C04, C05, C06, C07, C08, C09, C10, C11, C12, C13, C14, C15, C16, C17, C18, C19, C20, C21, C22, C23, C24, C25, C26, C30, C31, C32, C33, C34, C37, C38, C39, C40, C41, C45, C46, C47, C48, C49, C50, C51, C52, C53, C54, C55, C56, C57, C58, C60, C61, C62, C63, C64, C65, C66, C67, C68, C69, C70, C71, C72, C73, C74, C75, C76 |
| 2 | Leukemia | C91, C92, C93, C94, C95 |
| 2 | Lymphoma | C81, C82, C83, C84, C85, C88, C90, C96 |
| 3 | Moderate/severe liver disease | I85, K70.4, K72, K76.6 |
| 6 | Metastatic solid tumor | C77, C78, C79, C80 |
| 6 | AIDS | B20, B21, B22, B23, B24 |
| 1. **Ascertainment of other covariates**   Other covariates including smoking status, ethnicity, education level and income level were defined as follows:   \| **Traits** \| **Questions or descriptions in UK Biobank** \| **Category** \| \| --- \| --- \| --- \| \| Smoking status \| Smoking status \| Current: code for 2  Previous: code for 1  Never: code for 0  Unknown: code for -3 \| \| Ethnicity \| Ethnic background \| White: code for 1, 1001, 1002, 1003  Asian: code for 3, 3001, 3002, 3003, 3004, 5  Black: code for 4, 4001, 4002, 1003  Mixed: code for 2, 2001, 2002, 2003, 2004  Other: code for 6  Unknown: code for -3 or -1 \| \| Education level \| Qualifications \| College or university degree: code for 1,  A levels /AS levels /O levels /GCSEs / GSEs or equivalent: code for 2,3 or 4,  Other degree: code for 5 or 6,  Unknown: code for -7 or -3 \| \| Income level \| Average total household income before tax \| Ultra-high: code for 5  High: code for 4  Medium: code for 2 or 3  Low: code for 1  Unknown: code for -1 or -3 \| | | |

**6. Ascertainment of polygenic risk score for mild, moderate and severe COVID-19**

To avoid sample overlap, the GWAS summary statistics used here were derived from HGI database excluding participants from 23andMe and UK Biobank. 27,259 individuals from the matched cohort containing (40,479) were selected for calculating the polygenic risk score (PRS) of mild, moderate and severe COVID-19 according to the tutorial ^4^. PRS was constructed by aggregating the numbers of risk allele at each locus and weighted by the corresponding beta coefficient.

In brief, palindromic single nucleotide polymorphism (SNP) (i.e., SNPs with A/T or C/G at intermediate frequencies) in the base target (publicly available GWAS summary statistics of COVID-19 susceptibility, severity and hospitalization from the HGI database) were firstly removed to select valid SNPs for PRS ^4^. For target data (27,259 UKB participants), standard SNP-level quality control was performed as follows: SNPs with call rate >99% (less than 1% missing data), MAF>1%, and Hardy–Weinberg equilibrium (HWE) P > 1.0 × 10^−6 5^, which resulted in a total of 6,279,351 SNPs retained for following analysis.

In order to find “best-fitted” PRS of mild to severe COVID-19, we tested different PRS derived from different sets of SNPs in target data (Linkage Disequilibrium r^2^<0.1, and in the 250 kb region using 1000 Genomes projects as reference panel, passing with different p value thresholds at 0.00000001, 0.0000001, 0.000001, 0.00001, 0.0001, 0.001, 0.01, 0.05, 0.1, 0.2, 0.3, 0.4, and 0.5), by using logistic regression adjusted with age (continuous variables), gender (male/female) and principal components (PC) 1 to 20 (continuous variables) ^6^. The performances of different models were listed as follows:

| Threshold of P | Number of SNPs used in PRS | R^2^ of model | P | OR (95%CI) |
| --- | --- | --- | --- | --- |
| **Mild COVID-19** | | | | |
| 0.00000001 | 1407 | 0.001546 | 1.03 × 10^-19^ | 1.11 (1.09-1.14) |
| 0.0000001 | 1681 | 0.001789 | 1.91 × 10^-22^ | 1.12 (1.10-1.15) |
| 0.000001 | 2058 | 0.001684 | 3.45 × 10^-21^ | 1.12 (1.09-1.14) |
| 0.00001 | 3158 | 0.001449 | 2.02 × 10^-18^ | 1.11 (1.08-1.14) |
| 0.0001 | 6602 | 0.001446 | 2.64 × 10^-18^ | 1.11 (1.08-1.14) |
| 0.001 | 28194 | 0.001012 | 2.44 × 10^-13^ | 1.09 (1.07-1.12) |
| 0.01 | 177971 | 0.001019 | 2.06 × 10^-14^ | 1.09 (1.07-1.12) |
| 0.05 | 761697 | 0.001141 | 8.61 × 10^-15^ | 1.10 (1.07-1.12) |
| 0.1 | 1457336 | 0.001099 | 2.59× 10^-14^ | 1.10 (1.07-1.12) |
| 0.2 | 2793723 | 0.001250 | 5.05 × 10^-16^ | 1.10 (1.08-1.13) |
| 0.3 | 4097413 | 0.001191 | 2.36 × 10^-15^ | 1.10 (1.07-1.13) |
| 0.4 | 5382926 | 0.001169 | 4.15 × 10^-15^ | 1.10 (1.07-1.13) |
| 0.5 | 6660149 | 0.001161 | 5.14 × 10^-15^ | 1.10 (1.07-1.12) |
| **Moderate COVID-19** | | | | |
| 0.00000001 | 4334 | 0.000499 | 1.58 × 10^-7^ | 1.06 (1.04-1.09) |
| 0.0000001 | 4885 | 0.000445 | 7.03 × 10^-7^ | 1.06 (1.04-1.09) |
| 0.000001 | 5786 | 0.000501 | 1.59 × 10^-7^ | 1.06 (1.04-1.09) |
| 0.00001 | 7397 | 0.000500 | 1.68 × 10^-7^ | 1.06 (1.04-1.09) |
| 0.0001 | 11537 | 0.000481 | 2.90 × 10^-7^ | 1.06 (1.04-1.09) |
| 0.001 | 29086 | 0.000647 | 3.59 × 10^-9^ | 1.07 (1.05-1.10) |
| 0.01 | 147336 | 0.000596 | 1.42 × 10^-8^ | 1.07 (1.05-1.10) |
| 0.05 | 588432 | 0.000569 | 2.88 × 10^-8^ | 1.07 (1.04-1.09) |
| 0.1 | 1099671 | 0.000530 | 8.02 × 10^-8^ | 1.07 (1.04-1.09) |
| 0.2 | 2073502 | 0.000508 | 1.46 × 10^-7^ | 1.07 (1.04-1.09) |
| 0.3 | 3020495 | 0.000563 | 3.33 × 10^-8^ | 1.07 (1.04-1.09) |
| 0.4 | 3953005 | 0.000556 | 4.10 × 10^-8^ | 1.07 (1.04-1.09) |
| 0.5 | 4879548 | 0.000534 | 7.28× 10^-8^ | 1.07 (1.04-1.09) |
| **Severe COVID-19** | | | | |
| 0.00000001 | 3258 | 0.000198 | 5.65 × 10^-4^ | 1.04 (1.02-1.07) |
| 0.0000001 | 3787 | 0.000250 | 1.33 × 10^-4^ | 1.05 (1.02-1.07) |
| 0.000001 | 4879 | 0.000297 | 3.68 × 10^-5^ | 1.05 (1.03-1.07) |
| 0.00001 | 6837 | 0.000279 | 6.26 × 10^-5^ | 1.05 (1.02-1.07) |
| 0.0001 | 10713 | 0.000447 | 6.85 × 10^-7^ | 1.06 (1.04-1.09) |
| 0.001 | 27624 | 0.000449 | 1.79 × 10^-7^ | 1.06 (1.04-1.09) |
| 0.01 | 141872 | 0.000783 | 1.00 × 10^-10^ | 1.08 (1.06-1.11) |
| 0.05 | 577403 | 0.000462 | 4.97 × 10^-7^ | 1.06 (1.04-1.09) |
| 0.1 | 1090393 | 0.000488 | 2.50 × 10^-7^ | 1.06 (1.04-1.09) |
| 0.2 | 2083880 | 0.000561 | 3.55 × 10^-8^ | 1.07 (1.04-1.09) |
| 0.3 | 3062916 | 0.000589 | 1.69 × 10^-8^ | 1.07 (1.05-1.10) |
| 0.4 | 4027380 | 0.000621 | 7.37 × 10^-9^ | 1.07 (1.05-1.10) |
| 0.5 | 4987111 | 0.000614 | 8.74 × 10^-9^ | 1.07 (1.05-1.10) |

We found that PRS with a p threshold of 0.0000001 for mild COVID-19 explaining the highest phenotypic variance of 0.18%. The p threshold for moderate and severe COVID-19 were 0.001 and 0.01, which explained 0.065% and 0.078% phenotypic variance. Finally, 1681 SNPs for mild COVID-19, 29086 SNPs for moderate COVID-19 and 141872 SNPs for severe COVID-19 were retained for PRS calculation, by summing the reference alleles for each SNP weighted by the summary regression coefficient. The PRS was calculated using PLINK1.9 software ^5^.

**References**

1. Said MA, Verweij N, van der Harst P. Associations of combined genetic and lifestyle risks with incident cardiovascular disease and diabetes in the UK Biobank Study. *JAMA cardiology* 2018; **3:** 693-702.

2. Hou C, Hu Y, Yang H*, et al.* COVID-19 and risk of subsequent life-threatening secondary infections: a matched cohort study in UK Biobank. *BMC medicine* 2021; **19:** 1-10.

3. Quan H, Sundararajan V, Halfon P*, et al.* Coding algorithms for defining comorbidities in ICD-9-CM and ICD-10 administrative data. *Medical care* 2005**:** 1130-1139.

4. Choi SW, Mak TS-H, O’Reilly PF. Tutorial: a guide to performing polygenic risk score analyses. *Nature protocols* 2020; **15:** 2759-2772.

5. Reed E, Nunez S, Kulp D*, et al.* A guide to genome‐wide association analysis and post‐analytic interrogation. *Statistics in medicine* 2015; **34:** 3769-3792.

6. Li C, Chen Y, Chen Y*, et al.* The Causal Association of Irritable Bowel Syndrome with Multiple Disease Outcomes: A Phenome-Wide Mendelian Randomization Study. *Journal of Clinical Medicine* 2023; **12:** 1106.
