# Supplementary material for: The Time-Varying Impact of COVID-19 on the Acute Kidney Disorders: A Historical Matched Cohort Study and Mendelian Randomization Analysis
Source: Health Data Sci. 2024 Jul 15;4:0159. doi: 10.34133/hds.0159 (PMC11246837; doi:10.34133/hds.0159)
Supplement: Supplementary 1 — Supplementary Text Tables S1 to S6 [file hds.0159.f1.zip › Supplementary Table S1-identification of severe covid-19.docx]

Supplementary Table S1. Procedure definition of invasive or non-invasive mechanical ventilation or other respiratory support for severe COVID-19 patients.

| OPCS-4-codes | Procedure |
| --- | --- |
| E85.1 | Invasive ventilation |
| E85.6 | Continuous positive airway pressure |
| E85.2 | Non-invasive ventilation |
| X52.9 | Unspecified oxygen therapy |
| X52.8 | Other specified oxygen therapy |
| E85.8 | Other specified ventilation support |
| E85.9 | Unspecified ventilation support |
| E87.8 | Other specified oxygen therapy support |
| E87.9 | Unspecified oxygen therapy support |
