# Supplementary material for: The Time-Varying Impact of COVID-19 on the Acute Kidney Disorders: A Historical Matched Cohort Study and Mendelian Randomization Analysis
Source: Health Data Sci. 2024 Jul 15;4:0159. doi: 10.34133/hds.0159 (PMC11246837; doi:10.34133/hds.0159)
Supplement: Supplementary 1 — Supplementary Text Tables S1 to S6 [file hds.0159.f1.zip › Supplementary Table S2. Codes for kidney disease-0406.docx]

Supplementary Table S2. Codes for acute kidney disorder, chronic kidney disease and other kidney disease.

| **Kidney traits** | **ICD-9 codes** | **ICD-10 codes** | **OPCS-4 codes** |
| --- | --- | --- | --- |
| **Inclusion criteria of acute kidney disorder** | | | |
| Inpatient diagnosis/Death register | - | N00, N01, N10, N17, Y60.2, Y84.1, Z49.0, Z49.1, Z99.2 | - |
| Operations codes (OPCS-4) | - | - | X40.1, X40.3, X40.4, X40.5, X40.6, X40.7, X40.8, X40.9, X41.8, X41.9, X42.1, X42.8, X42.9 |
| **Exclusion criteria for congenital kidney disease** | | | |
| Inpatient diagnosis | 753 | Q60, Q61, Q62, Q63 | - |
