# Supplementary material for: The Time-Varying Impact of COVID-19 on the Acute Kidney Disorders: A Historical Matched Cohort Study and Mendelian Randomization Analysis
Source: Health Data Sci. 2024 Jul 15;4:0159. doi: 10.34133/hds.0159 (PMC11246837; doi:10.34133/hds.0159)
Supplement: Supplementary 1 — Supplementary Text Tables S1 to S6 [file hds.0159.f1.zip › supplementary Table S4. SMD within two groups-0406.docx]

Supplementary Table S4. Standard mean difference (SMD) between exposed and unexposed controls before and after inverse probability weights.

|  | SMD before weighting | SMD after weighting |
| --- | --- | --- |
| Age at index date | 0.01 | 0.006 |
| Sex | 0.006 | 0.001 |
| Ethnicity | 0.078 | 0.001 |
| Townsend deprivation index | 0.001 | 0.001 |
| BMI | 0.129 | < 0.001 |
| Smoking status | 0.011 | 0.002 |
| Education level | 0.054 | 0.001 |
| Income level | 0.040 | 0.001 |
| Hypertension at index date | 0.027 | 0.001 |
| Diabetes at index date | 0.095 | 0.001 |
| Charlson comorbidity index | 0.159 | 0.003 |
| CKD at index date | 0.078 | < 0.001 |

BMI, Body mass index; CKD, Chronic kidney disease
