# Supplementary material for: The Time-Varying Impact of COVID-19 on the Acute Kidney Disorders: A Historical Matched Cohort Study and Mendelian Randomization Analysis
Source: Health Data Sci. 2024 Jul 15;4:0159. doi: 10.34133/hds.0159 (PMC11246837; doi:10.34133/hds.0159)
Supplement: Supplementary 1 — Supplementary Text Tables S1 to S6 [file hds.0159.f1.zip › supplementary Table S6. sensitivity AKI-0406.docx]

| **Sensitivity analysis** | **No. of cases/1000 person-day** | | **Total** | | **The first week (Day 0 to 7)** | | **The second week (Day 8 to 14)** | | **The third week (Day 15 to 21)** | | **The fourth week (Day 22 to 28)** | |
| --- | --- | --- | --- | --- | --- | --- | --- | --- | --- | --- | --- | --- |
|  | **Exposed** | **Matched controls** | **HR (95%CI)** | ***P* value** | **HR (95%CI)** | ***P* value** | **HR (95%CI)** | ***P* value** | **HR (95%CI)** | ***P* value** | **HR (95%CI)** | ***P* value** |
| **Primary analysis ^a^** | 458/1.77 | 240/0.3 | 5.78 (4.84-6.91) | < 0.001 | 5.70 (4.73-6.89) | < 0.001 | 14.25 (6.35-31.97)) | < 0.001 | 4.11 (1.66-10.17) | 0.002 | 1.83 (0.58-5.83) | 0.30 |
| **Post weighting ^b^** | 458/1.77 | 240/0.3 | 5.78 (4.98-6.72) | < 0.001 | 4.88 (4.12-5.78) | < 0.001 | 10.06 (4.95-20.42) | < 0.001 | 3.58 (1.65-7.76) | 0.001 | 1.81 (0.63-5.15) | 0.27 |

Supplementary Table S6. Sensitivity analysis of COVID-19 on the risk incident acute kidney injury (AKI).

**^a^** Covariates adjusted in the model including age at index date, BMI, hypertension, diabetes, CKD, smoking status, education level, income level and Charlson comorbidity index.

^b^ Univariate Cox proportional hazard regression was performed after inverse probability weighting based on age at index date, sex, Twonsend deprivation index, BMI, smoking status, education level, income level, diabetes, hypertension, CKD and Charlson comorbidity index.
