# Supplementary material for: The Time-Varying Impact of COVID-19 on the Acute Kidney Disorders: A Historical Matched Cohort Study and Mendelian Randomization Analysis
Source: Health Data Sci. 2024 Jul 15;4:0159. doi: 10.34133/hds.0159 (PMC11246837; doi:10.34133/hds.0159)
Supplement: Supplementary 1 — Supplementary Text Tables S1 to S6 [file hds.0159.f1.zip › Supplementary Table S3-gwas summary data-20230613.doc]

Supplementary Table S3. GWAS summary statistics used for COVID-19

| Traits | Sample size (cases/controls) | Population | Consortium | URLs |
| --- | --- | --- | --- | --- |
| COVID-19 severity illed cases vs. population | 17472/725695 | All participants excluding 23andMe and UKB | THE COVID-19 Host Genetics Initiative (r7) | https://storage.googleapis.com/covid19-hg-public/freeze_7/results/20220403/leave_one_out/sumstats/COVID19_HGI_A2_ALL_leave_23andme_and_UKBB_20220403.tsv.gz (April 8, 2022, Release 7) |
| COVID-19 hospitalization vs. population | 40929/1924400 | All participants excluding 23andMe and UKB | THE COVID-19 Host Genetics Initiative (r7) | https://storage.googleapis.com/covid19-hg-public/freeze_7/results/20220403/leave_one_out/sumstats/COVID19_HGI_B2_ALL_leave_23andme_and_UKBB_20220403.tsv.gz (April 8, 2022, Release 7) |
| COVID-19 susceptibility vs. population | 143839/2357647 | All participants excluding 23andMe and UKB | THE COVID-19 Host Genetics Initiative (r7) | https://storage.googleapis.com/covid19-hg-public/freeze_7/results/20220403/leave_one_out/sumstats/COVID19_HGI_C2_ALL_leave_23andme_and_UKBB_20220403.tsv.gz (April 8, 2022, Release 7) |
